# Delayed diagnosis of extrapulmonary tuberculosis presenting as fever of unknown origin in an intermediate-burden country

**DOI:** 10.1186/s12879-018-3349-5

**Published:** 2018-08-28

**Authors:** Jeong-Han Kim, Eu Suk Kim, Kang-Il Jun, Hyun gul Jung, Ji Hwan Bang, Pyeong Gyun Choe, Wan Beom Park, Kyoung-Ho Song, Hong Bin Kim, Nam Joong Kim, Myoung-don Oh, Sang-Won Park

**Affiliations:** 1 Department of Internal Medicine, Boramae Medical Center, 20, Boramae-ro 5-gil, Dongjak-gu, Seoul, 07061 South Korea; 2 Department of Internal Medicine, Seoul National University College of Medicine, Seoul, Republic of Korea

**Keywords:** Extrapulmonary tuberculosis, Fever of unknown origin, FUO, Imaging study

## Abstract

**Background:**

Tuberculosis (TB), especially extrapulmonary tuberculosis (EPTB), is an important cause of fever of unknown origin (FUO) in TB-burdened areas. Little information is known about patients with EPTB with clinical features presenting as FUO and about the factor of delaying the diagnosis.

**Methods:**

We retrospectively analyzed EPTB patients who were referred with FUO at 3 university-affiliated hospitals over 8 years (2010–2017). The subjects were assigned to groups of early diagnosis and delayed diagnosis within 3 days of an initial comprehensive evaluation from the referral. Clinical and laboratory variables were compared between the groups.

**Results:**

A total of 95 patients with febrile EPTB were included. Localizing symptoms and/or signs suggestive of anatomy were identified in 62.1% of the patients. Concurrent lung involvement by TB was presented by 49.5% (47/95) of the patients, and only 23.4% of them showed typical findings of pulmonary TB on simple chest X-ray. Most of the patients showed abnormal lesions on cross-sectional CT (98.9%) and MRI (100%). The clinical variables and blood test results of patients were not significantly different between the two groups. The less typical imaging finding of EPTB on CT (38.5% vs. 79.0%) and MRI (37.5% vs. 79.0%) in the delayed diagnosis group was a risk factor for delayed diagnosis.

**Conclusion:**

Febrile EPTB referred as FUO showed nonspecific clinical manifestations. The active application of cross-sectional imaging tests according to clinical clues or randomly in the absence of local manifestations, combined with invasive diagnostic approaches even for atypical presentations may lead to an earlier diagnosis of febrile EPTB.

## Background

Fever of unknown origin (FUO) has been a challenging medical condition, even for infectious diseases specialists. As the rapid diagnosis of FUO is highly dependent on the expertise of medical staffs in charge and the technological support, the definition of FUO may be a matter of relativity. An increased awareness of common causes of FUO and advances in diagnostic assays has made the diagnosis of FUO easier than before [1–3]. However, infectious diseases still remain a top priority for FUO, and tuberculosis (TB) is one of the highly prevalent infectious diseases worldwide [2–4].

In South Korea, a country with an intermediate TB burden, TB is one of the differential diseases regularly considered in the evaluation of FUO. The proportion of TB in the final diagnosis of FUO was 19–27% in the 1990s, and this decreased to 8–11% in the 2000s, which still indicates TB as an important cause of FUO [5–8].

TB has a wide spectrum of clinical manifestations and consists of 80–85% pulmonary TB (PTB) and 15–20% extrapulmonary TB (EPTB) [9, 10]. Fever is one of the main manifestations in TB patients, present in 60–85% of PTB cases and 30–55% of EPTB cases [11–13]. PTB can be easily suspected by typical presentations, such as persistent cough, fever, weight loss and abnormal chest X-ray findings. However, cases of EPTB tend to present with atypical manifestations, which make it difficult to be suspected. In addition, EPTB frequently involves anatomical sites that are not easily accessible and require invasive procedures for diagnostic confirmation. For these reasons, EPTB is one of the differential diagnoses of FUO. However, little information is available about the clinical features of EPTB presenting as FUO and the reason why its diagnosis is delayed. As TB is a global problem, the characterization of EPTB presenting as FUO may be helpful for many clinicians.

## Methods

### Subjects and study design

**Fig. 1:**
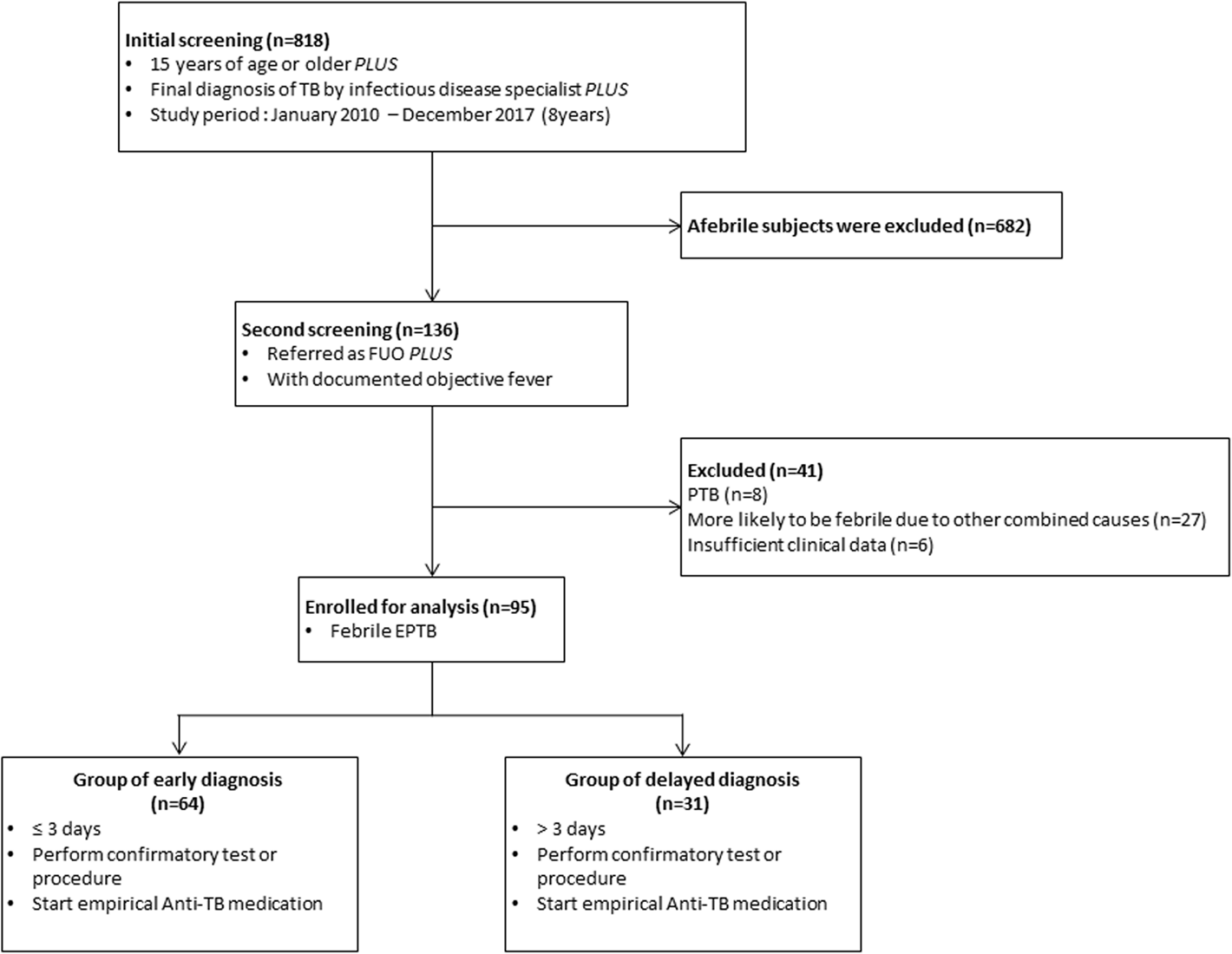
Study design. *EPTB* extrapulmonary tuberculosis, *FUO* fever of unknown origin, *PTB* pulmonary tuberculosis, *TB* tuberculosis

This retrospective case-series analysis was conducted at 3 Seoul National University (SNU)-affiliated hospitals (Boramae Medical Center, SNU Hospital and SNU Bundang Hospital). All patients who were ≥15 years old and had a final diagnosis of TB by infectious disease specialists during an 8-year period (2010–2017) were screened. Among them, febrile patients who were referred as having FUO and were confirmed to have objective fever at the time of referral were selected. PTB or patients who were estimated to have other causes of fever, such as concurrent other infections, drug-related fever or noninfectious diseases were excluded. Hence, only patients with febrile EPTB were included in the analysis (Fig. 1). Objective fever was defined as the highest daily body temperature ≥ 37.8 °C measured at the axilla [14]. The subjects were divided into groups of early and delayed diagnoses of EPTB according to the Durack and Street criteria for classic FUO (Fig. 1). Delayed diagnosis was defined as the failure to determine a proper diagnosis after 3 days of a comprehensive evaluation, including complete blood count, chemistry, urinalysis, cultures of blood and urine, simple chest X-ray, and abdominal computed tomography (CT) scan or ultrasonography [3, 15–17]. The early diagnosis group included patients who were diagnosed with EPTB ≤3 days from the initial FUO evaluation. The delayed diagnosis group included patients who were diagnosed with EPTB > 3 days from the initial FUO evaluation. The time of diagnosis was defined as the time of performing confirmatory tests or procedures or the time of starting empirical anti-TB drugs by clinical decision.

Clinical information of the subjects was collected via electronic medical records (EMR). We collected demographic variables, previous history of TB and comorbidities, including diabetes mellitus, hypertension, chronic kidney disease (estimated glomerular filtration rate by the Modification of Diet in Renal Disease formula: < 60 mL/min/1.73 m^2^), heart failure, hepatitis or cirrhosis of any cause, chronic obstructive lung disease, cerebrovascular accident with residual sequelae, solid organ malignancy, hematologic malignancy, autoimmune disease, solid organ transplantation and human immunodeficiency virus (HIV) infection. The information about the diagnosis and treatment of TB was included, such as the presence of localizing symptoms and signs that suggested infection or inflammation of a specific site, dates of variables, anatomic site of infection, laboratory test results, performance of radiologic and microbiologic tests, and clinical response to anti-TB medication.

### Definition of terms

Based on the composite reference standard, the diagnosis of TB was categorized into 3 groups: definite, probable and possible TB [18, 19]. Definite TB was defined as being culture-positive for *Mycobacterium tuberculosis* (MTB) or positive for both smear for acid-fast bacilli (AFB) and MTB-polymerase chain reaction (PCR) without culture results. Probable TB was defined as being negative for MTB culture but having clinical symptoms and radiologic findings suggestive of TB plus histologic or cytologic findings suggestive of TB (for example, granuloma with caseating necrosis in tissue, lymphocyte-dominant pleural effusion and ascites with high adenosine deaminase, lymphocyte-dominant high leukocyte count and increased protein/low glucose level in cerebrospinal fluid) or positive for AFB smear or positive for MTB-PCR. Possible TB was defined as being negative for MTB culture and other microbiologic tests but having clinical signs and/or symptoms suggestive of TB improved by empirical anti-TB medication. The treatment outcome was categorized into 4 groups according to the revised World Health Organization guidelines: success, failure, death and default [20]. TB involving the lungs or tracheobronchial tree was classified as PTB [21]. Involvement of any extrapulmonary site (for example, the peritoneum, lymph nodes, genitourinary tract, bone and joint, pleura, liver, bone marrow or meninges) was classified as EPTB. When EPTB and PTB concurrently existed, it was classified as EPTB [10, 13, 22]. Disseminated TB was defined as involvement of ≥2 noncontiguous sites and included miliary TB, bacteremic TB and the involvement of the liver or bone marrow [23]. A typical TB image finding was defined as a lesion for which TB could be suspected first depending on each anatomical site by radiologists [24].

### Statistical analysis

Continuous variables were compared using Student’s t-test, and categorical variables were compared using Pearson’s chi-square test or Fisher’s exact test. All tests of significance were 2-tailed, and *p* < 0.05 was considered significant. Statistical analyses were performed with STATA, version 12.0 (StataCorp LP, College Station, TX, USA).

## Results

### Baseline characteristics

A total of 95 patients with febrile EPTB were included. Fifty-three (55.8%) of the patients were male, and the median age was 56 (range: 15–88) years. Approximately half of the patients (47.4%) did not have any underlying comorbidities. The common comorbid diseases were hypertension (26.3%), diabetes mellitus (19.0%) and chronic kidney disease (19.0%). The median time of diagnosis after referral was 2 days (interquartile range: 1–5 days). Positive results of AFB smear and MTB-PCR or histologic/cytologic findings obtained from pulmonary or extrapulmonary specimens were the most common indications for initiation of TB treatment (82.1%), followed by a clinical decision (possible TB) (15.8%) and positivity by MTB culture (2.1%) (Table 1). The indirect drug susceptibility test was performed on 94 of 98 positive culture isolates, and 2 (2.0%) of them showed drug resistance: one with resistance to rifampin and quinolone (*n* = 1) and the other with resistance to streptomycin, ethambutol and p-amino-salicylic acid (n = 1).

**Table 1:** Baseline characteristics of febrile extrapulmonary tuberculosis patients referred with fever of unknown origin

Variables		Total (*n* = 95)	Group of early diagnosis (*n* = 64)	Group of delayed diagnosis (*n* = 31)	*p*-value
Median age, years (range)		56 (15–88)	51 (18–81)	64 (15–88)	0.128
Male sex, n (%)		53 (55.8)	37 (57.8)	16 (51.6)	0.568
Previous history of TB, n (%)		7 (7.4)	4 (6.3)	3 (9.7)	0.549
Comorbidity, n (%)	None	45 (47.4)	33 (51.6)	12 (38.7)	0.239
	Diabetes mellitus	18 (19.0)	8 (12.5)	10 (32.3)	0.021
	Hypertension	25 (26.3)	13 (20.3)	12 (38.7)	0.056
	Chronic kidney disease	18 (19.0)	9 (14.1)	9 (29.0)	0.081
	Heart failure	9 (9.5)	4 (6.3)	5 (16.1)	0.123
	Chronic liver disease	3 (3.2)	3 (4.7)	0	0.548
	Chronic obstructive lung disease	1 (1.1)	0	1 (3.2)	0.326
	CVA with residual sequelae	9 (9.5)	5 (7.8)	4 (12.9)	0.467
	Solid organ malignancy	2 (2.1)	2 (3.1)	0	1.000
	Hematologic malignancy	5 (5.3)	2 (3.1)	3 (9.7)	0.326
	Autoimmune disease	6 (6.3)	5 (7.8)	1 (3.2)	0.660
	Solid organ transplantation	2 (2.1)	2 (3.1)	0	1.000
	HIV-infected	4 (4.2)	3 (4.7)	1 (3.2)	1.000
Median fever duration before admission, days (IQR)		11 (0–29)	12.5 (0–25.5)	8 (0–31)	0.178
Median duration from admission to diagnosis, days (IQR)		2 (1–5)	1 (0–2)	8 (5–11)	<0.001
Median duration from anti-TB medication to the resolution of fever, days (IQR)		3 (2–5)	2 (2–11)	3 (2–4)	0.199
Median length of hospital stay, days (IQR)		14 (9–24)	12 (7–22)	21 (15–32)	0.037
Indication for the start of anti-TB medication, n (%)	MTB culture	2 (2.1)	2 (3.1)	0	0.695
	AFB smear, MTB-PCR, histopathology/cytology	78 (82.1)	51 (79.7)	27 (87.1)	
	Clinical decision	15 (15.8)	11 (17.2)	4 (12.9)	
Final diagnosis, n (%)	Definite TB	68 (71.6)	49 (76.6)	19 (61.3)	0.271
	Probable TB	23 (24.2)	13 (20.3)	10 (32.2)	
	Possible TB	4 (4.2)	2 (3.1)	2 (6.5)	
Treatment outcome, n (%)	Success	78 (82.1)	52 (81.3)	26 (83.9)	1.000
	Failure	0	0	0	
	Death	4 (4.2)	3 (4.7)	1 (3.2)	
	Default	13 (13.7)	9 (14.0)	4 (12.9)	

Abbreviations: *AFB* acid-fast bacilli, *CVA* cerebrovascular accident, *EPTB* extrapulmonary tuberculosis, *HIV* human immunodeficiency virus, *IQR* interquartile range, *PTB* pulmonary tuberculosis, *TB* tuberculosis

### Clinical findings

The clinical findings for febrile EPTB patients are shown in Table 2. Localizing symptoms and/or signs suggestive of diagnostic clues could be identified in 62.1% (59/95) of the patients. The common locations were bones and joints (*n* = 18), the cardiorespiratory system (*n* = 15) and the abdomen (*n* = 12). Anemia, leukocytosis, thrombocytopenia and thrombocytosis were observed in 64.2%, 22.1%, 14.7% and 12.6% of the patients, respectively. The mean value of C-reactive protein (CRP) was 10.2 mg/dL (range: 0.82–30.1 mg/dL).

**Table 2:** Clinical findings of febrile extrapulmonary tuberculosis patients referred with fever of unknown origin

Variables	Detail	Total (n = 95)	Group of early diagnosis (n = 64)	Group of delayed diagnosis (n = 31)	*P*-value
Localizing signs and/or symptoms, *n* (%)		59 (62.1)	42 (65.6)	17 (54.8)	0.310
Hematology	Hemoglobin (g/dL)	11.6 ± 2.1	11.9 ± 2.0	11.0 ± 2.4	0.050
	WBC (×1000/mm^3^)	7.7 ± 4.4	7.2 ± 3.6	8.7 ± 5.7	0.110
	Platelet (×1000/mm^3^)	259 ± 124	265 ± 123	247 ± 126	0.529
Chemistry	CRP (mg/dL)	10.2 ± 6.5	10.7 ± 6.9	9.0 ± 5.6	0.218
	Albumin (g/dL)	3.3 ± 0.5	3.3 ± 0.5	3.3 ± 0.5	0.889
	Alkaline phosphatase (IU/L)	143.2 ± 148.6	141.8 ± 169.5	145.9 ± 93.8	0.901
Lung involvement		47 (49.5)	34 (53.1)	13 (41.9)	0.306
Typical presentation of chest X-ray		11 (23.4)	11 (32.3)	0	0.014
Abnormal finding on CT scan		87/88 (98.9)	62/62 (100)	25/26 (95.2)	
Typical imaging findings of TB		60/88 (68.2)	49/62 (79.0)	11/26 (42.3)	<0.001
Anatomical distribution^a^					
Abdominopelvic region		34/52 (65.4)	26/34 (76.5)	8/18 (44.4)	
Chest		37/56 (66.1)	32/41 (78.0)	5/15 (33.3)	
Neck		3/5 (60.0)	2/3 (66.7)	1/2 (50.0)	
Abnormal finding on MRI		27/27 (100)	19/19 (100)	8/8 (100)	
Typical imaging findings of TB		18/27 (66.7)	15/19 (79.0)	3/8 (37.5)	0.037
Anatomical distribution^a^					
Musculoskeletal		12/21 (57.1)	10/14 (71.4)	2/7 (28.6)	
Brain		7/7 (100)	6/6 (100)	1/1 (100)	
Abnormal finding on PET-CT		6/6 (100)	1/1 (100)	5/5 (100)	

All values are the mean ± standard deviation or number (%), except where indicated otherwise

^a^The numbers of the typical imaging findings of TB (a) among cases in each anatomical distribution (b) were presented as ‘a/b (percentage)’

Abbreviations: *CRP* C-reactive protein, *CT* computed tomography, *MRI* magnetic resonance imaging, *PET-CT* positron emission tomography-computed tomography, *WBC* white blood cell

Concurrent pulmonary involvement of TB was observed in 47 patients (49.5%), and only 23.4% (11/47) of them showed typical findings of pulmonary TB on simple chest X-ray. Atypical imaging findings were more common in the delayed diagnosis group (*p* = 0.014). Atypical cases needed additional diagnostic tests such as chest CT scan, bronchoscopy and sputum culture to suspect TB, in addition to the evaluation of EPTB for the final diagnosis.

CT scans for EPTB prompted by local manifestations or routine evaluation were performed in 88 patients and showed abnormal focal lesions requiring further investigations to obtain diagnostic samples in 87 patients (98.9%). The common anatomic lesions identified on CT scan were as follows: miliary lung nodules (*n* = 22), mediastinal lymphadenopathy (*n* = 19), peritoneum and/or ascites (*n* = 17), lung parenchymal lesion (*n* = 15), intraabdominal lymphadenopathy (*n* = 13), liver and/or spleen (*n* = 12), pleura (*n* = 7) and paravertebral abscess (n = 7). Typical CT findings suggestive of TB were observed in 60 patients (68.2%). Magnetic resonance imaging (MRI) was performed in 27 patients and showed abnormal focal lesions, which consisted of musculoskeletal (*n* = 23) and brain (n = 7) locations in all patients. The common anatomic lesions identified on MRI scan were those of the thoracic or lumbar spine (*n* = 16), brain parenchyma (*n* = 5) and peripheral joint (*n* = 3). Typical MRI findings suggestive of TB were observed in 18 patients (66.7%). Fluorodeoxyglucose positron emission tomography (PFT)-CT was performed in 6 patients and showed hypermetabolic lesions suggesting active inflammation in all 6 patients. Focal lesions identified on CT scan and hypermetabolic lesions found on PET-CT were matched in 6 patients, and PET-CT additionally showed abnormal focal lesions that were not identified on CT scan in 2 patients.

### Features of early diagnosis group versus those of delayed diagnosis group

Among the 95 patients, 31 (32.6%) patients were classified into the group of delayed diagnosis, and the remaining 64 (67.4%) patients were classified into the group of early diagnosis. The proportion of localizing symptoms/signs was not significantly different between the two groups. The common anatomical locations of TB in the group of delayed diagnosis were disseminated infection (*n* = 9, 29.0%), miliary infection (*n* = 7, 22.6%), bone and joint infection (*n* = 3, 9.8%), mediastinal lymphadenopathy (*n* = 2, 6.4%), intraabdominal lymphadenopathy (n = 2, 6.4%), peripheral lymphadenopathy (n = 2, 6.4%) and peritonitis (n = 2, 6.4%). In the group of early diagnosis, the common anatomic locations were disseminated infection (*n* = 22, 34.3%), miliary infection (*n* = 21, 32.7%), peritonitis (*n* = 8, 12.5%), bone and joint infection (n = 3, 4.7%), pleurisy (n = 3, 4.7%) and mediastinal lymphadenopathy (n = 2, 3.1%). The anatomic distribution was also not significantly different between the two groups.

The epidemiological feature and results of laboratory blood tests were not significantly different between the two groups, except for diabetes mellitus (12.5% vs. 32.3%, *p* = 0.021) (Table 1 and Table 2). Length of hospitalization was longer in the group of delayed diagnosis (median: 21 days, *p* = 0.037). Patients in the group of delayed diagnosis showed less typical imaging findings suggestive of TB on CT scan (42.3% vs. 79.0%, *P* < 0.001) and MRI (37.5% vs. 79.0%, *p* = 0.037) than those in the group of early diagnosis (Table 2). The performance results of TB diagnostic assays using various clinical specimens were not significantly different between the two groups, and the performance was highest with the histologic or cytologic method (72.7% vs. 71.7%) and lowest with the AFB smear (15.2% vs. 23.0%). However, MTB culture positivity was marginally lower in the group of delayed diagnosis than in the group of early diagnosis (59.3% vs. 75.6%) (Table 3).

**Table 3:** Comparison of performances of diagnostic tests in tissues and clinical samples

Specimen type	No. (%) of positive specimens							
	Group of early diagnosis				Group of delayed diagnosis			
	AFB smear	MTB-PCR	Histology/cytology	MTB culture	AFB smear	MTB-PCR	Histology/cytology	MTB culture
Tissue								
Lymph node, cervical	2/5	4/5	4/5	2/4	0/4	3/4	3/3	2/2
Lymph node, non-cervical	2/7	5/7	7/7	3/4	2/5	4/5	4/5	0/3
Bone	2/9	8/9	5/8	8/9	0/4	2/4	2/4	3/4
Omentum	1/7	5/7	5/7	2/5	0/4	3/4	4/4	
Liver/spleen/bone marrow	0/4	2/4	1/3	2/4	3/8	6/8	5/7	0/3
Other soft tissue	5/7	6/7	4/7	4/5	2/3	3/3	2/3	2/2
Clinical samples								
Sputum	9/26	8/18		26/26	0/10	3/5		10/10
Bronchoscopic specimens	1/4	3/4		4/4				
CSF	0/4	1/4	0/4	2/4	0/2	1/2	0/2	1/2
Pleural fluid	0/7	1/7	6/7	2/7	0/2	0/2	2/2	0/2
Ascites	0/12	3/12	11/12	6/11	0/2	0/2	2/2	0/2
Other fluid	1/8	7/7		7/7	0/2	1/2	0/2	1/2
Total	23/100 (23.0)	53/91 (58.2)	43/60 (71.7)	68/90 (75.6)	7/46 (15.2)	26/41 (63.4)	24/33 (72.7)	19/32 (59.3)

Abbreviations: *AFB* acid-fast bacilli, *CSF* cerebrospinal fluid, *FUO* fever of unknown origin, *TB-PCR* tuberculosis polymerase chain reaction, *MTB Mycobacterium tuberculosis*

## Discussion

Our research question was why the diagnosis of EPTB referred as alleged FUO was difficult and how we could better diagnose EPTB presenting as FUO. Although South Korea is an intermediate TB-burdened country and doctors have many chances to care for EPTB patients, it is not uncommon that they misdiagnose febrile EPTB. In contrast to PTB, which manifests critical clues, such as respiratory symptoms or typical findings on chest X-ray, the diagnosis of EPTB depends on a high index of suspicion. Delay of the proper diagnosis may have a negative impact on the prognosis of individual treatment and can cause confusion in the differential diagnosis. Although the proportion of EPTB is relatively low, it remains steady not only in South Korea but also in areas with a low TB prevalence [21, 25].

Various hematologic profiles of TB have been reported in many parts of the world [12, 26–28]. Anemia of chronic disease is the most frequently encountered hematologic profile in TB and has been reported to have a prevalence of approximately 50–80%. The prevalence of other hematologic profiles, such as leukocytosis, thrombocytopenia and thrombocytosis, are reported to be approximately 10–20%. Increased alkaline phosphatase is a relatively common finding in a patient with TB, although the mechanism for this finding in TB is yet to be investigated [27, 29]. Our study produced laboratory profiles of febrile EPTB similar to those of previous reports. These laboratory parameters may not have diagnostic significance or play a predictive role in the diagnosis of febrile EPTB. A previous study reported that CRP elevation was presented in 63.1% of EPTB patients [12]. However, CRP elevation was observed in all of our febrile EPTB patients.

We could localize abnormal focal lesions using cross-sectional imaging tests such as CT scan and MRI in most cases. One patient showed no abnormal findings on CT, and this was a case of failure to identify bone abnormalities in abdominopelvic CT modality. The patient was finally diagnosed with TB spondylitis via musculoskeletal MRI. Because EPTB may have few symptoms/signs and laboratory findings that lead to suspicion of EPTB, cross-sectional imaging tests are critical for guiding further invasive approaches for diagnostic clinical specimens. We may use cross-sectional CT scan or MRI modalities according to the anatomic clues from patients, but even in the absence of any localizing symptoms/signs and any abnormal findings on simple chest X-ray, the blind use of CT scans for the chest and abdominopelvic region may be justifiable to evaluate FUO patients, especially in TB-burdened countries, considering that localizing symptoms/signs were identified in only 62.1% of study subjects in this study. PET-CT offered a supporting approach to identify the metabolic status of abnormal focal lesions and thus guide invasive approaches to the proper target. However, PET-CT findings alone did not distinguish infection from malignancy or inflammation. PET-CT may provide useful information for identifying anatomic sites in the evaluation of FUO [30–32]. There were several case series that reported the clinical role of FDG PET-CT in the diagnosis of EPTB [33–35]. PET-CT was also helpful for the diagnosis of six patients in our study. Although 49.5% of EPTB patients had concurrent lung involvement, simple chest X-ray was not a sufficient screening tool to lead to a TB diagnosis because only 23.4% of them showed typical TB findings on simple chest X-ray. However, further evaluation of atypical lung lesions may enhance the characterization of TB lung lesions.

There were no differences in the epidemiologic and laboratory variables between the two groups. The group of delayed diagnosis showed less typical TB imaging findings. Patients with typical imaging presentation on chest X-ray might be less likely to have a delayed TB diagnosis. The less typical imaging findings on CT or MRI might draw less attention from doctors and might have delayed the clinical decision to apply TB diagnostics or start empirical TB treatment. As the performance of diagnostic assays for TB was not significantly different between the two groups, the microbiologic factor might not have a significant effect on delayed diagnosis. Rather, the less typical imaging findings might be associated with a lower positivity of MTB culture in the group of delayed diagnosis. A possible explanation for this may be that EPTB in this group had a lower focal bacillary burden and less formation of localizing inflammatory mass. In contrast, EPTB in the group of early diagnosis might have a higher focal bacillary burden and inflammatory mass effect, leading to the presentation of more overt diagnostic features.

This study has a few limitations. First, the retrospective design itself might not provide sufficient clinical results. However, the sample size of this study may be sufficient for a retrospective review. Second, as we only included patients referred to infectious diseases specialists, febrile EPTB patients referred to other specialties might be excluded, and our subjects thus might not represent the febrile EPTB patient population as a whole.

## Conclusion

In summary, EPTB referred as FUO showed nonspecific clinical manifestations, and cross-sectional imaging tests, such as CT scan or MRI guided by localizing symptoms/signs or a routine protocol, were critical for detecting diagnostic clues of TB. Active consideration of early cross-sectional imaging tests in febrile patients combined with the invasive acquisition of diagnostic clinical specimens may lead to earlier diagnosis of febrile TB. Furthermore, the less typical imaging findings for EPTB on cross-sectional imaging tests should not be concluded as being non-TB because the atypical presentations may delay the diagnosis of TB. Finally, as the performances of EPTB diagnostic tests are variable, a combination of the best tests according to the specific situation of the patient must be applied.

## Abbreviations

AFB: acid-fast bacilli
CRP: C-reactive protein
CT: computed tomography
EMR: electronic medical record
EPTB: extrapulmonary tuberculosis
FUO: fever of unknown origin
HIV: human immunodeficiency virus
MRI: magnetic resonance imaging
MTB: *Mycobacterium tuberculosis*
PCR: polymerase chain reaction
PET: positron emission tomography
PTB: pulmonary tuberculosis
SNU: Seoul National University
TB: tuberculosis

## References

[1] Efstathiou SP, Pefanis AV, Tsiakou AG, Skeva II, Tsioulos DI, Achimastos AD (2010). Fever of unknown origin: discrimination between infectious and non-infectious causes. Eur J Intern Med.

[2] Kucukardali Y, Oncul O, Cavuslu S, Danaci M, Calangu S, Erdem H (2008). The spectrum of diseases causing fever of unknown origin in Turkey: a multicenter study. Int J Infect Dis.

[3] Hayakawa K, Ramasamy B, Chandrasekar PH (2012). Fever of unknown origin: an evidence-based review. Am J Med Sci.

[4] Onal IK, Cankurtaran M, Cakar M, Halil M, Ulger Z, Dogu BB (2006). Fever of unknown origin: what is remarkable in the elderly in a developing country?. J Inf Secur.

[5] Oh MD, Peck KR, Song YW, Choe KW (1993). A clinical study on 55 patients with fever of undetermined origin. Korean J Infect Dis.

[6] Oh MD (1998). Fever of undeterminde origin: common diseases. J Korean Med Assoc.

[7] Kim YK, Kim MS, Lee KS, Huh AJ, Yeom JS, Hong SK (2001). A comparison of causes of fever of unknown origin between the 1980s and the 1990s. Korean J Med.

[8] Kee SY, Jo YM, Kim JY, Choi WS, Jeong HW, Jung SJ, et al. Etiology of adult patients with fever of unknown origin (FUO) observed in a university hospital in Korea from 1998-2003. Infect Chemother. 2005;37(3):127-32.

[9] Dheda K, Barry CE, Maartens G (2016). Tuberculosis. Lancet.

[10] Peto HM, Pratt RH, Harrington TA, LoBue PA, Armstrong LR (2009). Epidemiology of extrapulmonary tuberculosis in the United States, 1993-2006. Clin Infect Dis.

[11] Kiblawi SS, Jay SJ, Stonehill RB, Norton J (1981). Fever response of patients on therapy for pulmonary tuberculosis. Am Rev Respir Dis.

[12] Yoon HJ, Song YG, Park WI, Choi JP, Chang KH, Kim JM (2004). Clinical manifestations and diagnosis of extrapulmonary tuberculosis. Yonsei Med J.

[13] Gonzalez OY, Adams G, Teeter LD, Bui TT, Musser JM, Graviss EA (2003). Extra-pulmonary manifestations in a large metropolitan area with a low incidence of tuberculosis. Int J Tuberc Lung Dis.

[14] Mackowiak PA, Wasserman SS, Levine MM (1992). A critical appraisal of 98.6 degrees F, the upper limit of the normal body temperature, and other legacies of Carl Reinhold august Wunderlich. JAMA.

[15] Arnow PM, Flaherty JP (1997). Fever of unknown origin. Lancet.

[16] Knockaert DC, Vanderschueren S, Blockmans D (2003). Fever of unknown origin in adults: 40 years on. J Intern Med.

[17] Bleeker-Rovers CP, Vos FJ, de Kleijn EM, Mudde AH, Dofferhoff TS, Richter C (2007). A prospective multicenter study on fever of unknown origin: the yield of a structured diagnostic protocol. Medicine (Baltimore).

[18] Hawkridge A, Hatherill M, Little F, Goetz MA, Barker L, Mahomed H (2008). Efficacy of percutaneous versus intradermal BCG in the prevention of tuberculosis in south African infants: randomised trial. BMJ.

[19] Vadwai V, Boehme C, Nabeta P, Shetty A, Alland D, Rodrigues C (2011). Xpert MTB/RIF: a new pillar in diagnosis of extrapulmonary tuberculosis?. J Clin Microbiol.

[20] Eurosurveillance editorial team Collective. WHO revised definitions and reporting framework for tuberculosis. Euro Surveill. 2013;18(16):20455. 23611033

[21] ECDC. Tuberculosis surveillance and monitoring in Europe. 2013. https://ecdc.europa.eu/sites/portal/files/media/en/publications/Publications/Tuberculosis-surveillance-monitoring-2013.pdf. Accessed 16 June 2018.

[22] Kruijshaar ME, Abubakar I (2009). Increase in extrapulmonary tuberculosis in England and Wales 1999-2006. Thorax.

[23] Wang JY, Hsueh PR, Wang SK, Jan IS, Lee LN, Liaw YS (2007). Disseminated tuberculosis: a 10-year experience in a medical center. Medicine (Baltimore).

[24] Joshua Burrill CJW, Bain G, Conder G, Hine AL, Misra RR (2007). Tuberculosis: a radiologic review. Radiographics.

[25] Lee JY (2015). Diagnosis and treatment of extrapulmonary tuberculosis. Tuberc Respir Dis (Seoul).

[26] Gonzalez Saldana N, Macias Parra M, Hernandez Porras M, Gutierrez Castrellon P, Gomez Toscano V, Juarez Olguin H (2014). Pulmonary tuberculous: symptoms, diagnosis and treatment. 19-year experience in a third level pediatric hospital. BMC Infect Dis.

[27] Mert A, Arslan F, Kuyucu T, Koc EN, Ylmaz M, Turan D (2017). Miliary tuberculosis: Epidemiologicaland clinical analysis of large-case series from moderate to low tuberculosis endemic country. Medicine (Baltimore).

[28] Kassa E, Enawgaw B, Gelaw A, Gelaw B. Effect of anti-tuberculosis drugs on hematological profiles of tuberculosis patients attending at University of Gondar Hospital, Northwest Ethiopia. BMC Hematol. 2016;16:1. 10.1186/s12878-015-0037-1 PMC4706672 26751690

[29] Onur ST, Iliaz S, Iliaz R, Sokucu S, Ozdemir C (2016). Serum alkaline phosphatase may play a role in the differential diagnosis of sarcoidosis and tuberculosis. Int J Clin Exp Med.

[30] Palestro CJ, Love C (2018). Nuclear medicine imaging in fever of unknown origin: the new paradigm. Curr Pharm Des.

[31] Becker W, Meller J (2001). The role of nuclear medicine in infection and inflammation. Lancet Infect Dis.

[32] Bleeker-Rovers CP, Vos FJ, Mudde AH, Dofferhoff ASM, de Geus-Oei LF, Rijnders AJ (2007). A prospective multi-centre study of the value of FDG-PET as part of a structured diagnostic protocol in patients with fever of unknown origin. Eur J Nucl Med Mol Imaging.

[33] Lefebvre N, Argemi X, Meyer N, Mootien J, Douiri N, Sferrazza-Mandala S (2017). Clinical usefulness of (18)F-FDG PET/CT for initial staging and assessment of treatment efficacy in patients with lymph node tuberculosis. Nucl Med Biol.

[34] Hou S, Shen J, Tan J (2017). Case report: multiple systemic disseminated tuberculosis mimicking lymphoma on 18F-FDG PET/CT. Medicine (Baltimore).

[35] Machackova H, Pilka R, Losse S, Zurkova M, Lostakova V, Tichy T (2017). A case report of peritoneal tuberculosis: the diagnostic role of PET/CT and laparoscopy. Ceska Gynekol.

